# Psychopharmacology in children with genetic disorders of epigenetic and chromatin regulation

**DOI:** 10.1186/s11689-025-09605-9

**Published:** 2025-04-24

**Authors:** Sophia Lenz, Ajilan Sivaloganathan, Sarah J. Goodman, Cheryl Cytrynbaum, Jesiqua Rapley, Emma Canning, Danielle Baribeau

**Affiliations:** 1 Autism Research Centre, Holland Bloorview Kids Rehabilitation Hospital, Toronto, Canada; 2 Institute of Medical Sciences, University of Toronto, Toronto, Canada; 3 Faculty of Medicine, University of Toronto, Toronto, Canada; 4 The Hospital for Sick Children, Toronto, Canada; 5 Department of Molecular Genetics, University of Toronto, Toronto, Canada; 6 Carleton University, Ottawa, Canada; 7 Autism Research Centre, Bloorview Research Institute, Holland Bloorview Kids Rehabilitation Hospital, 150 Kilgour Rd, Toronto, ON M4G 1R8 Canada; 8 Department of Psychiatry, University of Toronto, Toronto, Canada; 9 Institute of Health Policy, Management and Evaluation, University of Toronto, Toronto, Canada

**Keywords:** Rare genetic neurodevelopmental disorders, Autism, Psychopharmacology, Behavioural disorders, Epigenetic regulation

## Abstract

**Objective:**

Hundreds of rare genetic variants associated with autism or intellectual disability have been identified, and many impact genes known to have a primary epigenetic/chromatin regulatory function. The objective of this study was to examine and compare behavioural profiles and longitudinal psychotropic treatment patterns in children with epigenetic/chromatin variants, other rare variants impacting neurodevelopment, or no known genetic condition.

**Methods:**

Using electronic medical records from a pediatric psychopharmacology program for children with autism or intellectual disability, we compared clinical characteristics, longitudinal psychotropic medication profiles and side effects between those with and without a rare genetic variant, and by variant subtype [epigenetic/chromatin regulation or other variant].

**Results:**

A total of 331 children attended 2724 unique medical visits between 2019 and 2022, with a mean of 8 follow-up visits over 3.4 years. Nine children (3%) had variants in epigenetic/chromatin regulatory genes (EC), twenty-three children (7%) had other rare genetic variants (OTH), and the rest had no reported variant (NR, *n* = 299, 90%). Those with a rare genetic variant (EC or OTH) were more likely to have an intellectual disability and had a greater number of co-occurring physical health conditions (*p* < 0.01). Overall, 66% of psychotropic medications were continued for ≥ 3 visits, while 26% were discontinued. Rates of psychotropic polypharmacy, medication patterns, behavioural challenges, and co-occurring developmental diagnoses were similar between genetic groups. Analyses uncorrected for multiple comparisons suggested those with genetic variants were more likely to experience drowsiness/sedation as a side effect (EC 33%, OTH 35%, NR 16%, *p* < 0.05); weight gain as a side effect was also higher in the epigenetic/chromatin group (EC 50% vs OTH 11%).

**Conclusion:**

Genetic classification of neurodevelopmental disorders (NDDs) may help anticipate treatment tolerability; additional prescribing considerations may be needed for those with rare variants. Current psychotropic prescribing practices do not differ across rare genetic NDD subgroups.

**Supplementary Information:**

The online version contains supplementary material available at 10.1186/s11689-025-09605-9.

## Introduction

Autism spectrum disorder and intellectual disability (ID) are childhood onset neurodevelopmental disorders (NDDs) that affect 1–3% of the population [1, 2]. Approximately 1/3 of autistic individuals have a co-occurring ID. [2] Children and youth with autism or ID are at 2 to tenfold greater risk of experiencing a co-occurring mental health condition in childhood (e.g., depression or anxiety) [3–5], and challenging behaviors such as irritability, aggression and self-injury can affect up to 50% [6, 7]. Beyond behavioural and environmental supports, psychotropic medications are often prescribed in efforts to manage co-occurring symptoms and behaviours. Estimates of psychotropic medication use in children with autism or ID range from 30–60%, and psychotropic polypharmacy (taking 2 or more medications concurrently) is common [8, 9]. Clinical wisdom and prior data suggest that people with autism and/or ID may be more vulnerable to side effects from psychotropic medications [10], and guidelines suggest clinicians ought to “start low and go slow” when prescribing for this population [11]. There are justified concerns about overprescribing [9], yet there are limited published clinical data [12, 13], and no biomarkers, to guide psychotropic medication choice in NDDs.

At the same time, with increased access to genome wide sequencing [14, 15], an underlying rare genetic variant impacting neurodevelopment (i.e., a rare genetic neurodevelopmental disorder) can now be clinically identified in 10% to 50% of those diagnosed with autism or ID [14]. Thus far, over 1000 different rare genetic variants impacting neurodevelopment have been identified, [16–18]; this genetic heterogeneity poses a challenge for neuroscience, clinical research, and precision medicine [19]. Existing data suggest that children with rare genetic NDDs have higher medical complexity, including increased subspecialist care and a greater number of co-occurring physical health concerns, compared to children with NDDs without an underlying genetic diagnosis [20–22]. It is also established that those with NDD-associated variants have substantially increased likelihood of being diagnosed with a developmental and/or neuropsychiatric condition compared to the the general population [23]. However, there are significant gaps in phenotype reporting regarding mental health and behavioural outcomes in rare genetic case descriptions which rely heavily on early childhood or cross-sectional data [24]. With the exception of a few better characterized genetic conditions, (e.g., 22q11.2 or 3q29 deletions and schizophrenia, *SHANK3* and bipolar disorder, psychotic disorders in Prader Willi Syndrome) [25–29], for most rare variants, the rapid rate of genomic discovery has outpaced the generation of clinical evidence. As such, very little is known about how or whether a rare genetic diagnosis may help anticipate mental health challenges or shape care planning and treatment approach in individuals with NDDs [24, 28].

Given the high genetic heterogeneity in NDDs, the potential to subgroup rare NDD variants by function or molecular pathway to help guide clinical care is appealing, as is beginning to emerge in epilepsy [30], and has been investigated regarding autism phenotypes [21, 31]. Emerging evidence suggests that most rare genetic NDD variants coalesce into a few common underlying molecular pathways [e.g., synaptic scaffolding and signalling—‘synaptopathies’ [32], ion channels—‘channelopathies’ [33], and disorders of epigenetic and chromatin regulation – ‘chromatinopathies’ [34–36]]. There are sometimes clinical similarities among conditions within the same molecular subgroup (e.g., [33]). Disorders of epigenetic and chromatin regulation in particular share the common clinical features of growth abnormalities, intellectual disability, a tendency towards overweight/obesity, high rates of autism, ADHD, and high behavioural care needs [34, 37, 38]. Recognized neurodevelopmental syndromes resulting from variants in epigenetic machinery and/or chromatin regulation genes include Coffin-Siris syndrome, Rett syndrome, Cornelia de Lange syndrome, Kabuki syndrome, and Kleefstra syndrome, among others.

Regarding psychotropic medication management in rare genetic NDDs, some data suggest that individuals with rare variants may be more sensitive to side effects [28, 39, 40]. For example, those with 22q11.2 deletion syndrome were more likely to experience serious adverse effects from clozapine, compared to those with schizophrenia without a 22q11.2 deletion [39]. Individuals with Phelan-McDermid syndrome are reported to have poor tolerance to psychostimulants and selective serotonin reuptake inhibitors [41]. In the epigenetic/chromatin disorders specifically, there are little published data. A high proportion of individuals with ARID1B-related Coffin-Siris syndrome discontinued treatment due to tolerability concerns in a recent trial of low dose clonazepam [42]. On the other hand, in Kleefstra syndrome, a case series suggests higher dose olanzapine may be especially helpful for insomnia and regression, and experts advise against “starting low and going slow” in this context [40]. Overall, the relevance of a rare genetic diagnosis, and/or the molecular pathway affected by a rare variant, with respect to psychotropic medication management for most children with NDDs is unknown, and is a recognized clinical and research priority for this community [24, 37, 43].

In this study, using a large clinical cohort of children with autism or ID receiving medication management for behavioural or mental health concerns, we identified a subgroup of children with rare genetic NDDs and divided them into those with variants in single genes that are known to have a primary epigenetic regulatory function, those with other rare variants subtypes, and those without a known genetic variant. We then 1) compared the clinical characteristics of children with and without rare variants regarding co-occurring physical, developmental, and behavioural conditions, and explored differences between rare variant subtypes (epigenetic/chromatin vs. other). We hypothesized that the rates of co-occurring physical and behavioural health concerns and intellectual disability would be higher for those with rare variants, and that there would be higher rates of obesity and intellectual disability in those with epigenetic variants specifically. Next, we then examined psychotropic medication treatment patterns, including polypharmacy, medication classes tried, medication discontinuation rates and side effects reported, across the three subgroups. We hypothesized that children with rare variants broadly would have higher rates of medication discontinuation, more reported side effects and lower rates of polypharmacy compared to those without rare variants.

## Methods

### Study design

We conducted a retrospective chart review study from medical records related to a large clinical program (the Psychopharmacology Program, *n* = 6 physicians) providing longitudinal care to children and youth with autism or intellectual disability and behavioural and/or mental health challenges that have not responded to behavioural, environmental and psychosocial interventions and at least one psychopharmacologic medication trial. This multidisciplinary tertiary clinical program provides service to a geographic region comprising 15 million residents, and sees an average of 200 unique patients annually, with follow-up visits scheduled every 3–6 months. This chart review study was approved by the Research Ethics Board at Holland Bloorview Kids Rehabilitation Hospital (REB #0592).

### Study population

We identified a sample of patients who attended one or more psychopharmacology medical visits between January 1, 2019 and January 1, 2022.

### Chart abstraction

We identified all consultation and follow-up visit medical records documented by a physician or nurse practitioner for each included patient. These were manually reviewed by trained chart abstractors and key clinical, demographic, medical, and treatment variables (see below) were entered into a de-identified database via semi-structured survey. Each abstractor co-reviewed 5 patient charts with another abstractor, and then reliability was estimated for survey scoring. Discrepancies were discussed, and a subsequent set of 5 charts were then co-reviewed until percent agreement across items reached 85%. Once reliability was achieved, charts were reviewed by a single abstractor.

### Variables of interest

Clinician documentation in the Psychopharmacology Program took place using semi-structured electronic medical record templates. Presence of a rare genetic disorder, (i.e., a pathogenic or likely pathogenic variant associated with neurodevelopmental impact) was defined through any documented history of such in the medical record; children without mention of a genetic diagnosis at any visit were included in the ‘no reported genetic diagnosis’ group. Individual variants were classified into those with a primary epigenetic regulatory function**,** or ‘other’, through manual variant pathway analysis, considering 1) published literature, 2) Fahrner and Bjornsson’s list of disease causing components of epigenetic machinery [34, 37], 3) a recently published epigenetic and chromatin gene list in NDDs [36], and 4) variant descriptions in OMIM. Other variables extracted included demographic characteristics, medical conditions, and psychiatric and behavioural symptoms (e.g., diagnoses of autism, ADHD, intellectual disability, types of behavioural concerns).

Psychotropic medication history was derived from the current medication reconciliation lists recorded in each medical visit note by the treating clinician. We examined psychotropic medication use across five classes (antipsychotics, psychostimulants, antidepressants, alpha adrenergic agonists, anticonvulsants taken for a behavioural indication, and sedatives/sleep aids). We did not examine anticonvulsant medications used for seizures or other medical disorders (e.g., pain). For each child, we examined their history of medications tried by class, as well as continuation and discontinuation rates for medication trials while followed in clinic. Regarding history of medications tried, we included both those tried prior to clinic intake (reviewed at the initial consultation), as well as those taken during longitudinal follow-up. Medication history by class is reported per individual (i.e., the number of individuals with a lifetime history of a medication trial in the class out of the number of total individuals). Regarding medications continued and discontinued, we restricted these analyses to children who attended three or more follow-up visits in the Psychopharmacology Program. We defined medication continuation as a child having continued a medication across three or more consecutive visits. We defined medication discontinuation as a child taking a medication at one or more visit followed by two consecutive visits where the medication was stopped or no longer included on their medication list. Medication continuation and discontinuation rates are reported out of the total number of medication trials as the denominator (therefore, a single individual may have contributed multiple medication trials per class). Regarding side effects, we examined reports from medical records for individuals who attended at least one follow-up visit, thereby restricting the analyses to active side effect reporting, not delayed recall regarding past medication trials.

### Analyses

Descriptive statistics (means, medians) and univariate analyses (Chi-squared test, Fisher’s exact test) were used to compare demographic and clinical data between children with a rare genetic NDD vs. without, and by variant subtype. We were underpowered given small sample sizes to compare medication patterns by genetic subgroups; means and proportions are displayed to convey trends, with differences between groups of > 15% indicated by an asterisk.

## Results

A total of 331 children/youth attended one or more medical visits through the Psychopharmacology Program between 2019 and 2022 (mean of 3.4 years of follow-up, standard deviation (SD) of 3.1 years). All of their longitudinal visits were reviewed (2724 visits total). A total of 9 individuals with genetic variants in epigenetic/chromatin regulatory genes (3%) (Table 1 ) and 23 individuals with other genetic variants (7%) were identified (Table 1). The remaining 299 individuals (90%) had no reported genetic variant.

**Table 1:** Definition of variant subgroups

**Variants in single genes that are known to have a primary epigenetic regulatory function (*n*=9)**	**Other genetic diagnoses identified (*n*= 23)**	
ARID1B SMARCA2 EHMT1 MECP2 duplication MECP2 deletion SMC3 NSD1 SETD2 SPEN (55)	KIAA2022 SCN1A 16p13.1 microduplication 22q11.2 Deletion^a^(*n*=3) OPHN1 Klinefelter syndrome (56)^b^ 22q13 deletion^a^ Trisomy 14 mosaicism SHANK3 GRIN1	Waardenburg syndrome type 2A 15q13.1 deletion 8p23 translocation and partial deletion Xp22.3 microduplication Fragile X premutation (57)^b^ Chromosome 17 copy number variant CDKL5 Phenylketonuria (PKU) Tuberous sclerosis complex, TSC2 mutation (*n*=2)

Classification based on the gene list published by Fahrner, Bjornsson et al., [34, 37], and the curated gene list by Valencia et al [36]. Table 1 is classified and annotated based on currently known methylation alterations; however, it is likely that additional variants with methylation alterations will be identified in future, especially for copy number variants**.** We note that some variants categorized as “other” do have published epigenetic signatures in blood, presumably due to the presence of a gene with epigenetic regulatory function in the CNV (^a^), and that there are methylation changes noted with sex chromosome aneuploidy [44] and fragile X [45] (^b^)

As hypothesized, there was a higher rate of intellectual disability (56% vs 31%) and co-occurring medical conditions in those with rare genetic diagnoses broadly; however, rates of ADHD, aggressive behaviour, self-injurious behaviour, and crisis service use did not differ between groups with and without a rare genetic diagnosis (Table 2). Of 331 children/youth, 237 (72%) identified aggressive behaviour as a top symptom target. As hypothesized, there was a higher rate of obesity (78% vs 22%), and trends towards more intellectual disability (66% vs. 52%) in those with epigenetic variants compared to other variants (Table 3). Duration and intensity of treatment was the same across groups, with an average of 8–9 follow-up visits over 3–4 years of follow-up (Tables 2 and 3).

**Table 2:** Clinical characteristics comparing children with and without a rare genetic variant

	**Any genetic variant**	**No genetic variant reported**	***p***
n	32 (10%)	299 (90%)	
**Demographic characteristics**			
Female Sex, N(%)	9 (28%)	48 (16%)	0.1
Age at first visit, Mean (SD)	11.0 (3.1)	10.6 (3.1)	0.5
Number of follow-up visits, Mean (SD)	8.2 (5.0)	8.2 (6.3)	0.9
Follow-up time in years, Mean (SD)	3.8 (3.2)	3.4 (3.1)	0.5
**Co-occurring developmental and psychiatric conditions**			
Intellectual Disability, N (%)	18 (56%)	92 (31%)	0.007*
Autism diagnosis, N (%)	26 (81%)	274 (94%)	0.09
ADHD diagnosis, N (%)	16 (50%)	171(57%)	0.5
Non-speaking, N (%)	11 (34%)	68 (23%)	0.2
Past crisis service use for behaviour, N (%)	4 (13%)	47 (16%)	0.8
Aggressive behaviour, N (%)	22 (69%)	215 (72%)	0.7
Self-injurious behaviour, N (%)	14 (44%)	140 (47%)	0.8
**Medical history**			
Number of subspecialist physicians, Mean (SD)	3.3 (2.6)	1.2 (1.4)	< 0.001
Number of physical health conditions, Mean (SD)	1.6 (0.9)	1.1 (0.5)	< 0.001
Seizures, N (%)	13 (40%)	34 (11%)	< 0.001
Obesity, N (%)	12 (38%)	84 (28%)	0.3
Asthma, N (%)	2 (6%)	12 (4%)	0.6
GERD, N (%)	7 (22%)	7 (2%)	< 0.001*
Chronic constipation, N (%)	2 (6%)	16 (5%)	0.7

*P*-values from Fisher’s exact test or Chi-squared for counts/proportions, means/medians are compared with t-test (for normally distributed data) or Mann-Whitney U test (for non-parametric distributions). Results are reported uncorrected. GERD: gastroesophageal reflux disease

**Table 3:** Comparison of clinical characteristics by rare variant subtype

	**Genetic variant in epigenetic regulator**	**Other** **genetic variant**	***p*-value**
n	9 (3%)	23 (7%)	
**Demographic characteristics**			
Female Sex, N(%)	4 (44%)	5 (22%)	0.2*
Age at first visit, Mean (SD)	12.0 (2.9)	10.5 (3.1)	0.2
Number of follow-up visits, Mean (SD)	9.3 (5.9)	7.8 (4.7)	0.4
Follow-up time in years, Mean (SD)	3.7 (2.7)	3.8 (3.4)	0.9
**Co-occurring developmental and psychiatric conditions**			
Intellectual Disability, N (%)	6 (66%)	12 (52%)	0.7
Autism diagnosis, N (%)	7 (78%)	19 (83%)	0.9
ADHD diagnosis, N (%)	3 (33%)	13 (54%)	0.4*
Non-speaking, N (%)	3 (33%)	8 (35%)	0.9
Past crisis service use for behaviour, N (%)	1 (11%)	3 (13%)	0.9
Aggressive behaviour, N (%)	6 (66%)	16 (70%)	0.9
Self-injurious behaviour, N (%)	3 (33%)	11 (48%)	0.6*
**Medical history**			
Number of subspecialist physicians, Mean (SD)	3.6 (2.4)	3.2 (2.7)	0.5
Number of physical health conditions, Mean (SD)	1.8 (1.1)	1.6 (0.8)	0.6
Seizures, N (%)	4 (44%)	9 (39%)	0.9
Obesity, N (%)	7 (78%)	5 (22%)	0.006*
Asthma, N (%)	1 (11%)	1 (4%)	0.5
GERD, N (%)	1 (11%)	6 (26%)	0.6*
Chronic constipation, N (%)	1 (11%)	1 (4%)	0.5

*P*-values from Fisher’s exact test or Chi-squared for counts/proportions, means/medians are compared with t-test (for normally distributed data) or Mann-Whitney U test (for non-parametric distributions). Results are reported uncorrected. *Indicates group differences >15%. GERD: gastroesophageal reflux disease

Rates of psychotropic polypharmacy did not differ between those with and without a rare genetic diagnosis, or by variant subtype (Tables 4 and 5). The average child seen in clinic took up to 2–3 medications concurrently during follow-up; the most tried medication classes for the entire cohort were psychostimulants (72%), antipsychotics (69%), and sedative/sleep aids (66%). Data suggested that children with rare genetic conditions may be less likely to try an antidepressant agent (34% vs. 52%, *p* = 0.07), but were potentially more likely to try an alpha agonist (78% vs. 61%, *p* = 0.09). There were no differences in overall medication discontinuation rates, with 25% of those with a rare variant and 26% of those without, discontinuing a medication during follow-up. Atomoxetine was the most discontinued medication (40%) overall.

**Table 4:** Comparison of psychotropic medication treatment patterns between children with and without a rare genetic NDD variant

	**Any genetic variant**	**No genetic variant reported**	*p*
n total (%)	32 (10%)	299 (90%)	
n attended 1 or more follow-up visits (% of group)	27 (84%)	243 (81%)	
n attended 3 or more follow-up visits (% of group)	25 (78%)	228 (76%)	
**Polypharmacy**			
Max number of agents used concurrently, Mean (SD)	2.9 (1.3)	2.9 (1.1)	0.7
Max number of agents used concurrently, Median (IQR)	2 (2)	3 (2)	
**Medication classes ever tried per individual**^**a**^			
Antipsychotic trial	21 (65%)	208 (69%)	0.8
Stimulant/atomoxetine trial	21 (65%)	217 (72%)	0.7
Antidepressant trial	11 (34%)	158 (52%)	0.07*
Alpha agonist trial	25 (78%)	183 (61%)	0.09*
Anticonvulsant trial	5 (16%)	27 (9%)	0.2
Sedative-hypnotic or sleep aid trial	19 (59%)	196 (66%)	0.6
**Medication trials discontinued during follow-up**^**b**^			
Any medication trial discontinued	27/107 (25%)	224/852 (26%)	0.9
** Discontinued antipsychotic trials**	4/22 (18%)	59/203 (29%)	
Risperidone	3/10 (30%)	25/87 (29%)	
Aripiprazole	1/7 (14%)	17/66 (26%)	
** Discontinued stimulant/atomoxetine trial**	7/22 (32%)	47/158 (30%)	
Amphetamine class	2/2 (33%)	17/57 (30%)	
Methylphenidate class	3/11 (27%)	19/75 (23%)	
Atomoxetine	2/5 (40%)	11/26 (40%)	
** Discontinued alpha agonist trial**	5/21 (23%)	23/150 (15%)	
Clonidine	3/14 (21%)	14/77 (18%)	
Guanfacine XR	2/7 (29%)	9/73 (12%)	*
** Discontinued antidepressant trial**	4/13 (23%)	34/140 (24%)	
Sertraline	2/8 (25%)	12/49 (24%)	
** Discontinued sedative-hypnotic or sleep aid**	5/19 (26%)	48/164 (29%)	
Melatonin	4/10 (40%)	22/111 (20%)	*
**Side effects per individual during follow-up**^**c**^			
Any side effect report	17/27 (63%)	130/243 (53%)	0.5
Headache	0/27 (0%)	6/243 (2%)	0.9
Abdominal Pain	1/27 (3%)	19/243 (8%)	0.7
Constipation	2/27 (7%)	3/243 (1%)	0.07
Nausea/vomiting	2/27 (7%)	6/243 (2%)	0.2
Weight gain	6 (22%)	66/243 (27%)	0.7
Weight Loss	2 (7%)	18/243 (7%)	0.9
Irritability	2 (7%)	17/243 (7%)	0.9
Drowsiness/sedation	11 (41%)	48/243 (20%)	0.02
Appetite decrease	2 (7%)	23/243 (9%)	0.8
Appetite increase	7 (26%)	61/243 (25%)	0.9

*P*-values from Fisher’s exact test or Chi-squared for counts/proportions, means/medians are compared with t-test (for normally distributed data) or Mann–Whitney U test (for non-parametric distributions). Results are reported uncorrected. *Indicates group differences > 15%

a) Ever tried was defined as a patient who reported a past medication trial with an agent in the defined class as clinic intake, OR who trialed a medication in that class during clinic follow-up. Results are reported per patient, therefore a patient who tried three antipsychotic medications would contribute only one count to this outcome

b) Medication discontinuation rates were examined among patients who attended three or more follow-up visits in clinic. We defined medication discontinuation as a patient who took a medication at any point during follow-up care, who then discontinued that medication on two subsequent consecutive visits. Medication class discontinuation rates are reported per unique medication trial; therefore a single individual may have contributed multiple distinct medication trials to this outcome

c) Side effects were collected for patients who attended more than 1 follow-up visits, therefore restricting them to those reported during active medication trials (as opposed to retrospective reporting on past medication trials at clinic intake). Side effects are reported per patient. Therefore, a patient who reported headache as a side effect across multiple visits or medication trials would contribute only once to this outcome

^*^ > 15% difference between groups

**Table 5:** Comparison of psychotropic medication treatment patterns by variant subtype

	**Genetic variant in epigenetic regulator**	**Other genetic variant**	** > 15% difference**
n total	9	23	
n attended 1 or more follow-up visits	8	19	
n attended 3 or more follow-up visits	7	18	
**Polypharmacy**			
Max number of medications used concurrently during follow-up (mean (SD), median (IQR))	3.0 (1.3) 3 (2)	2.8 (1.4) 2 (2)	
**Medication classes ever tried per individual**^**a**^			
Antipsychotic trial	6 (66%)	15 (65%)	
Stimulant/atomoxetine trial	7 (78%)	15 (65%)	
Antidepressant trial	3 (33%)	8 (35%)	
Alpha agonist trial	7 (78%)	18 (78%)	
Anticonvulsant trial	3 (33%)	2 (9%)	*
Sedative-hypnotic or sleep aid	5 (55%)	14 (61%)	
**Medication trials discontinued during follow-up**^**b**^			
Antipsychotic trial	1/6 (17%)	3/16 (19%)	
Stimulant/atomoxetine trial	0/5 (0%)	5/14 (36%)	*
Alpha agonist trial	1/5 (20%)	4/16 (25%)	
Antidepressant Trial	0/4 (0%)	4/9 (25%)	*
Sedative-hypnotic or sleep aid	1/5 (20%)	5/15 (33%)	
**Side effects during follow-up (> 1 visit)**^**c**^			
Any side effect report	6 (75%)	11 (58%)	*
Headache	0 (0%)	0 (0%)	
Abdominal pain	1 (13%)	0 (0%)	
Constipation	0 (0%)	2 (10%)	
Nausea/ vomiting	1 (12.5%)	1 (5%)	
Weight gain	4 (50%)	2 (11%)	*
Weight Loss	1 (12.5%)	1 (5%)	
Irritability	0 (0%)	2 (11%)	
Drowsiness/ sedation	3 (38%)	8 (42%)	
Appetite decrease	0 (0%)	2 (11%)	
Appetite increase	2 (25%)	5 (26%)	

a) Ever tried was defined as a patient who reported a past medication trial with an agent in the defined class as clinic intake, OR who trialed a medication in that class during clinic follow-up. Results are reported per patient, therefore a patient who tried three antipsychotic medications would contribute only one count to this outcome

b) Medication discontinuation rates were examined among patients who attended three or more follow-up visits in clinic. We defined medication discontinuation as a patient who took a medication at any point during follow-up care, who then discontinued that medication on two subsequent consecutive visits. Medication class discontinuation rates are reported per unique medication trial; therefore a single individual may have contributed multiple distinct medication trials to this outcome

c) Side effects were collected for patients who attended more than 1 follow-up visits, therefore restricting them to those reported during active medication trials (as opposed to retrospective reporting on past medication trials at clinic intake). Side effects are reported per patient. Therefore, a patient who reported headache as a side effect across multiple visits or medication trials would contribute only once to this outcome

Rates of side effects were similar between those with (63%) and without (54%) a rare genetic condition (*p* = 0.5). Those with rare genetic variants had a higher rate of drowsiness/sedation as a side effect (41% vs. 20%, *p* = 0.02) (Table 4). Consistent with this, a greater proportion of children with rare genetic conditions discontinued guanfacine XR (29% vs 12%) and melatonin (40% vs. 20% during follow-up). Medication treatment patterns were similar between those with genetic disorders of epigenetic regulation and those with other rare variant subtypes. Differences between groups suggested those with epigenetic/chromatin variants were more likely to be prescribed an anticonvulsant for a behavioural indication during clinic follow-up (33% vs. 9%), and were potentially less likely to discontinue a stimulant or antidepressant (0% vs. 36% and 0% vs. 25%, respectively). Individuals reporting one or more side effects during follow-up were higher (75% vs 58%) in the epigenetic/chromatin group (Table 5), primarily due to increased reports of weight gain (50% vs 11%).

Of the overall cohort, 66% of medications initiated were continued across three visits or more (Supplemental Table 1). The most continued medications overall were clonidine (77%), guanfacine XR (78%), melatonin (73%) and sertraline (73%). Patterns suggested children with rare genetic conditions were perhaps more likely to continue clonidine (79%) as opposed to guanfacine XR (57%), while there were no such differences in the group without a rare genetic variant (77% and 78%).

## Discussion

We examined a cohort of children and youth with autism or intellectual disability receiving pharmacotherapy for behavioural/psychiatric symptoms through a large clinical program. Children with rare genetic NDDs had higher rates of medical complexity and intellectual disability than children with NDDs without a known genetic variant. We did not identify differences in the rates of co-occurring behavioural challenges or other developmental differences between those with and without a rare genetic condition. Psychotropic medication treatment patterns including rates of polypharmacy were similar across children with variants in single genes known to have a primary epigenetic regulatory function, other genetic NDDs, and NDDs with no known genetic disorder. While underpowered, potential differences in medication side effects by genetic subgroup emerged which merit further study, including higher rates of drowsiness/fatigue for those with any genetic disorder, and potentially increased weight gain in those epigenetic variants.

As hypothesized, the presence of rare genetic variants was associated with higher prevalence of intellectual disability in our cohort, as per prior studies [21, 22]. However, the observation that rare genetic variants were not significantly associated with the prevalence of other developmental or behavioural outcomes/phenotypes, such as ADHD, autism, communication abilities, crisis service use, or aggressive or self-injurious behaviour is important. This challenges prior notions that certain types of behaviours (e.g., self-injurious) may enable more targeted testing, instead supporting guidelines calling for genome wide testing approaches in NDDs [14].

Medication continuation and discontinuation rates were informative as a potential proxy for effectiveness and tolerability. For a child with autism or intellectual disability with at least one prior psychotropic medication trial, our data suggest that approximately 2/3rds of subsequent medication trials under expert care are likely to be sufficiently effective and tolerated to be continued for 9–12 months. While underpowered, our data could be consistent with a hypothesis that rare genetic variants/mechanisms broadly may explain part of the elevated side effect risk associated with NDDs, which if replicated, could have added clinical utility for prescribers. Medication continuation and discontinuation rates were consistent with the potentially increased reports of drowsiness/fatigue in those with rare variants, given the increased discontinuation of potentially sedating agents in those with rare variants (guanfacine XR, melatonin). The finding that children with rare genetic conditions were potentially less likely to take an antidepressant agent could reflect challenges with identifying mood and anxiety symptoms in children with intellectual disability [46], or reflect diagnostic overshadowing by physical health complexity.

For those with epigenetic and chromatin disorders specifically, most patients attending a specialized epigenetic and chromatin clinic had a neurodevelopmental disability, much of the visit time was focused on assessment and management of behaviour and developmental concerns, and a need for infrastructure for medication management was identified [37]. Our data suggest that the psychopharmacological management of this population at present is not dissimilar from children with other developmental or genetic disorders, although side effect profiles may require some unique consideration, and could justify subspecialist behavioural health care (e.g., [47]). Weight gain and sedation were prevalent side effects, which if replicated, have the potential to impact treatment decision making. We also note that a high proportion (7/9, 78%) of children with epigenetic/chromatin variants in our sample had a clinical history of obesity documented in the chart at intake; whether an epigenetic or chromatin variant increases the risk of weight gain from psychotropic medications beyond the risk associated with pre-existing overweight/obesity is unclear. Recent data suggest elevated insulin resistance and high triglycerides in some epigenetic/chromatin disorders (e.g., EHMT1) [48], and that epigenetic regulation is a potential mechanism in the pathophysiology of metabolic diseases (e.g., diabetes, dyslipidemia) in the general population [49]. Given the known metabolic side effects with atypical antipsychotics [50], added caution and monitoring in those with these conditions during treatment may be merited.

Results of this study should be considered in light of several limitations. Findings may not generalize to, nor represent the general autism or ID populations, given the specialized nature of the clinic supporting children with high behavioural and mental health care needs who have not responded to prior treatments and community programs. Our sample sizes for those with rare genetic variants were small as is expected with rare disorders, and analyses are uncorrected. Findings should be considered preliminary/hypothesis generating and in need of replication. Genetic diagnoses (10%) were also likely under captured in this cohort compared to expected yields with genome wide sequencing [14, 15]. Public funding for genome wide sequencing in autism or ID as a first tier test became available in our region in 2025, therefore it is likely that the true prevalence of rare conditions in the ‘no reported variant’ group is higher, and that single gene disorders would have been disproportionately ascertained for children with additional clinical features beyond neurodevelopmental differences. Children with no mention of a genetic diagnosis in their medical record were grouped with the “no variant” group, which risks misclassification bias, although this would bias away from detecting differences between groups. We used medication continuation and discontinuation as a proxy for effectiveness/tolerability, but were not able to differentiate specific reasons for discontinuation from this dataset. We note the Psychopharmacology Program is structured such that children who are behaviourally improved or stable transition out of active follow-up, back to their referring provider or to a transitional care program, therefore medications discontinued during follow-up would primarily represent those with tolerability/effectiveness concerns. Side effects were captured by clinician documentation of such in the progress note, and were not systematically assessed beyond routine clinical care. We also did not systematically examine trajectories of weight gain, although this will be a focus of future work.

In summary, given increased access to high resolution genetic technologies, the rising rates of genetic diagnoses [14, 15], and the justified concerns about psychotropic polypharmacy in autism and ID [8], data are needed to help individualize care for those with rare genetic conditions. Results suggest there is added complexity with psychotropic medications for those with rare genetic NDDs, including more medical comorbidities and potential additional concerns with tolerability, but that current prescribing practices are similar for those with and without a genetic condition. Preliminary data suggest children and youth with epigenetic and chromatin disorders may benefit from added vigilance regarding sedation and metabolic side effects during treatment with psychotropic medications for behavioural or mental health concerns.

## Supplementary Information

Supplementary Material 1.

## Data Availability

Data are from a clinical chart review; requests for de-identified data queries can be directed to the corresponding author.
