# Supplementary material for: Psychopharmacology in children with genetic disorders of epigenetic and chromatin regulation
Source: J Neurodev Disord. 2025 Apr 24;17:21. doi: 10.1186/s11689-025-09605-9 (PMC12023381; doi:10.1186/s11689-025-09605-9)
Supplement: Supplementary file 1 — Supplementary Material 1. [file 11689_2025_9605_MOESM1_ESM.docx]

**Psychopharmacology in children with genetic disorders of epigenetic and chromatin regulation**

**Supplemental Materials**

**Supplemental Table 1: Medication Trials Continued For At least Three visits During Follow-up by Genetic Subgroup**

|  | Epigenetic regulator | Other variant | Any genetic variant | No genetic variant |
| --- | --- | --- | --- | --- |
| n total individuals (%) | 9 | 23 | 32 | 299 |
| n individuals who attended 3 or more follow-up visits | 7 | 18 | 25 | 228 |
| **Any medication continued** | 22/28 (79%) | 49/79 (62%) | 71/107 (66%) | 544/852 (64%) |
| **Antipsychotic trial** | 5/6 (83%) | 11/16 (69%) | 16/22 (72%) | 122/203 (60%) |
| Risperidone | 3/3 (100%) | 4/7 (57%) | 7/10 (70%) | 56/87 (64%) |
| Aripiprazole | 2/3 (67%) | 4/4 (100%) | 6/7 (86%) | 44/66 (67%) |
| **Stimulant/ atomoxetine trial** | 4/5 (80%) | 8/14 (57%) | 12/19 (63%) | 99/158 (62%) |
| Amphetamine class | 3/4 (75%) | 2/4 (50%) | 5/8 (62%) | 37/67 (55%) |
| Methylphenidate class | 2/3 (67%)* | 3/8 (38%)* | 5/11 (45%)* | 49/75 (65%)* |
| Atomoxetine | - | 3/5 (60%) | 3/5 (60%) | 13/26 (50%) |
| **Alpha agonist trial** | 3/5 (60%) | 12/16 (75%) | 15/21 (71%) | 116/150 (77%) |
| Clonidine | 2/3 (67%) | 9/11 (82%) | 11/14 (79%) | 59/77 (77%) |
| Guanfacine XR | 1/2 (50%) | 3/5 (60%) | 4/7 (57%)* | 57/73 (78%)* |
| **Antidepressant trial** | 4/4 (100%)* | 4/9 (44%)* | 8/13 (61%) | 89/139 (64%) |
| Sertraline | 2/2 (100%) | 3/6 (50%) | 5/8 (62%) | 36/49 (73%) |
| **Anticonvulsant trial** | 1/1 (100%) | 1/2 (50%) | 2/3 (67%) | 4/9 (44%) |
| **Sedative-hypnotic or sleep aid** | 3/5 (60%) | 9/15 (60%) | 12/20 (60%) | 99/164 (60%) |
| Melatonin | 2/2 (100%) | 4/8 (50%) | 6/10 (60%)* | 81/111 (73%)* |

Medication continuation rates were examined among patients who attended three or more follow-up visits in clinic. We defined medication continuation as a patient who took a medication at any point during follow-up care and continued this medication for at least three consecutive visits. Medication class continuation rates are reported per unique medication trial; therefore a single individual may have contributed multiple distinct medication trials to this outcome.

* Group differences of interest, exceeding 15%
